# IL1β, IL-6, and TNF-α cytokines cooperate to modulate a complicated medical condition among COVID-19 patients: case-control study

**DOI:** 10.1097/MS9.0000000000000679

**Published:** 2023-04-26

**Authors:** Shalaw Sardar Faraj, Paywast Jamal Jalal

**Affiliations:** Department of Biology, College of Science, University of Sulaimani, Sulaymaniyah, Iraq

**Keywords:** Biomarker, IL-1β, IL-6, proinflammatory cytokine, SARS-CoV-2, TNF-α

## Abstract

The cytokine storm is a complication related to SARS-CoV-2 infection worldwide. This study aimed to address the level of three cytokines which were interleukin-1-beta (IL-1β), interleukin-6 (IL-6), and tumour necrosis factor-alpha (TNF-α), with different blood parameters to the formation of cytokine storm or any complication among COVID-19 patients.

**Method and material:**

A total of 104 serum samples were included for this purpose, and they were divided into three categories the healthy control group (*n*=30), mild COVID-19 patients (*n*=23), and severe cases of COVID-19 patients (*n*=51). The cytokine concentration was measured by enzyme-linked immunosorbent assays (ELISA). Serum ferritin, C-reactive protein (CRP) levels, and erythrocyte sedimentation rate were also evaluated and compared with the concentration of the proinflammatory cytokines.

**Result:**

The data analysis showed a significant relationship between the serum IL-6 level with serum ferritin and CRP and the progression to the severity of SARS-CoV-2 infection. The IL-6 level was increased in mild COVID-19 patients and was significantly elevated in severe COVID-19 patients. Patients in the severe group had significantly higher serum ferritin, CRP, and erythrocyte sedimentation rate levels than those in the mild and healthy groups. The IL-1β and TNF-α were not significantly different in the groups compared with the healthy control group.

**Conclusion:**

This study revealed that the proinflammatory cytokines and biochemical laboratory tests are promising biomarkers for detecting the severity of COVID-19 cases.

## Introduction

Highlights

- Coronavirus disease 2019 (COVID-19), which is brought on by the SARS-CoV-2 coronavirus, is a serious pandemic of the twenty-first century. The disease’s virulent tentacles have spread around the world, with unknown consequences and effects.
- The cytokine storm that results from the unchecked and increased release of proinflammatory cytokines and immune suppression that define advanced COVID-19 syndrome.
- The severity of the viral infection and mortality rate are both positively correlated with the unchecked and dysregulated release of inflammatory and proinflammatory cytokines.
- The release of several proinflammatory cytokines, including TNF-α, IL-1, and IL-6, causes the lung’s alveolar cells to attract macrophages, T, and B cells, which triggers an inflammatory response.

The severe acute respiratory syndrome coronavirus 2 (SARS-CoV-2) is the cause of the coronavirus disease of 2019 (COVID-19), which also results in explosive outbreaks and waves of morbidity and mortality that pose significant public health risks^1^. It is currently the third highly pathogenic beta coronavirus to cause severe human illness in the previous 20 years^2^. SARS-CoV-2 seems contagious, has spread more quickly worldwide, and infects upper and lower respiratory tracts^3^. Epithelial and immune cells interact through cell-to-cell contacts, and cytokine signalling controls viral propagation^4^. A small number of SARS-CoV-2-infected people may exhibit mild to severe COVID-19 symptoms. However, the majority may remain asymptomatic^1^. It has been illustrated that the immune system is a significant player in the pathogenesis of COVID-19 because the causative virus (ЅΑRЅ-CoV-2) can induce dysregulated innate and adaptive immune responses that are ultimately associated with widespread damage to tissues and organs^5^.

The predominant immunopathological mechanism driving a more severe clinical course in COVID-19 cases is the cytokine storm, which is also the cause of mortality^6^. When SARS-CoV-2 infection occurs, large amounts of proinflammatory cytokines, commonly referred to as cytokine storms, are released; these cytokines include interleukin-6 (IL-6), interleukin-1-beta (IL-1β), interleukin-10 (IL-10), interleukin-18 (IL-18), interleukin-4 (IL-4), interleukin-33 (IL-33), Interferon-gamma (IFN)-γ, and tumour necrosis factor-alpha (TNF-α)^4^. Increases in proinflammatory cytokines, including IL-1β, IL-6, and TNF-α, are indicators of illness progression and an approaching cytokine storm^7^.

Along with reverse transcription polymerase chain reaction, several biomarkers are employed to diagnose COVID-19. C-reactive protein (CRP) and serum ferritin are two examples of such biomarkers. Ferritin and CRP function as acute-phase reactants and produce cytokines and cytokine storms^8^. Despite the clinical questions, erythrocyte sedimentation rate (ESR) is another parameter frequently utilized as a cheap and accessible test in routine laboratory patient workups^9^. According to clinical evidence, elevated ferritin levels, which typically range from (18–350) ng/ml in serum, are associated with COVID-19 severity^7^. Ferritin levels are greater than 400 ng /ml and may be 1.5–5.3 times greater in severe cases than mild ones^7^. Although CRP levels are elevated in COVID-19 patients, studies have demonstrated a clear association between illness severity and prognosis: survivors (Moderate and Severe) had median CRP values of about 40 mg/l, whereas non-survivors (Critical) had median rates of 125 mg/l^10^.

In this study, we aimed to compare cytokines and disease severity levels between healthy control groups and mild and severe COVID-19 patients. Serum ferritin, CRP levels, and ESR were the additional biomarkers examined.

## Method and material

The work has been reported in line with the STROCSS criteria^11^. Moreover, this research was retrospectively registered to the scientific committee and ethically approved. It was formally accepted with approval from the College of Medicine’s Ethics Committee at the University of Sulaimani. Ethic Permission Number: 75 and the date 19/6/2022 by the College of Medicine at the University of Sulaimani ethic committee.

### Patients characteristics

The current research is a case-control study carried out on 104 Kurdish participants in Iraq, ages between (23 and 90 y), between (June 2021 and December 2021); the sample collection was done at the Laboratory of Shahid Hemin Teaching Hospital, Shorsh Teaching Hospital and Blood Bank Centre of Sulaymaniyah. The practical was carried out at the hospital laboratories, a private Laboratory and a College of science/ Biology department at the University of Sulaimani.

The Control group comprises 30 patients (Table 1). Control samples were obtained from healthy individuals who showed no clinical symptoms and had not interacted with the affected individuals. Furthermore, they do not carry the hepatitis B virus, hepatitis B core, hepatitis C virus, or HIV. Moreover, the serum ferritin, ESR, and CRP tests which are markers of inflammation, all demonstrated average results.

**Table 1:** Demographic information of various COVID-19 patients groups and healthy controls

Group	Frequency percentage, *n* (%)	Male	Female
Healthy	30 (28.8)	28	2
Mild	23 (22.1)	7	16
Severe	51 (49.0)	27	24
Total	104 (100.0)	62	42

The patients with COVID-19 were confirmed by reverse transcription polymerase chain reaction test, and both Mild and severe cases (Table 1) were separated based on the clinical study and haematological and biochemical tests. Mild/moderate cases: mild are those which are not hypoxic and without evidence of pneumonia; moderate is with clinical signs of pneumonia (fever, cough) but no symptoms of severe pneumonia (oxygen saturation≥90%). Severe cases: admitted to intensive care unit due to severe hypoxia (oxygen saturation<90%)^12^. Depending on their condition, the COVID-19 patients received treatment by WHO recommendations, which may have included basic analgesics like paracetamol and antivirals (e.g. remdesivir and favipiravir). Broad-spectrum antibiotics were administered to patients with a bacterial co-infection such as (e.g. meropenem and levofloxacin). Additionally, the patients got supportive therapies (e.g. vitamin C, vitamin D, and zinc), anticoagulants such as (clexane), and anti-inflammatory drugs like (dexamethasone). Oxygen was also given in severe situations, and upon admission, an ICU, in particular, was made available for urgent care.

### Haematological and biochemical test

All biochemical tests were measured by Cobas c311 analyzer (Roche Diagnostics) and according to the standard protocols. The ESR (average 0–20 mm/h) was calculated by the Westergren method.

### Enzyme-linked immune sorbent assay (ELISA)

The level of various serum cytokines, including TNFα, IL-1β, and IL-6, were measured by ELISA using Quantikine kits Bioassay Technology Laboratory (BT-lab China) according to the manufacturer’s protocol and the colours’ intensity was measured in a wavelength of 450 nm. The Sensitivity of each kit includes 1.52 ng/l, 10.07 pg/ml, and 1.03 ng/l, respectively.

### Statistical analysis

Using GraphPad Prism version 8.0, statistical analysis was carried out. The one-way ANOVA test, the Kruskal–Wallis test, the *t*-test, Post Hoc Tests (Tukey HSD), and the Mann–Whitney U test were applied for the analysis of the data. The Spearman or Pearson correlation coefficient was used to determine the correlation between two continuous variables. Within a 95% confidence interval, statistical analysis was performed. A *P* value of 0.05 was used to determine significance.

## Results

### No significant relation between the proinflammatory ratio and sex observed

In the presented study, no comparison did between control and infected patients with COVID-19 based on sex because the number of males and females was not equal. However, the comparison between males and females among both mild and severe cases based on sex showed that more males were infected with SARS-CoV-2 than females. A total of 74 COVID‐19 patient groups were included in this study; the mild group had 7 (6.7%) males and 16 (15.4%) females, while the severe group recruited 27(26%) males and 24 (23%) females (Table 1).

The median age was 62 years old (23–90). Cytokine and biochemical tests showed differences elevated between sexes among the groups of COVID-19. There was no significant difference between sex in the concentration levels of the three biomarkers (ferritin, CRP, ESR) and cytokines in the COVID-19 patient group, as shown in Table 2. This study indicated no difference between males and females in the side production of proinflammatory cytokine and other inflammatory markers among COVID-19 patients. Both had the same clinical effect on the patient.

**Table 2:** The comparison of cytokine among SARS-CoV-2 patients based on sex

Marker	Sex	*N*=74	Mean±SD	*P* value
IL-6 ng/l	Male	34	151.02±59.19	0.72
	Female	40	156.19±65.34	
IL1β ng/l	Male	34	52.31±51.71	0.63
	Female	40	58.05±50.95	
TNF α ng/l	Male	34	179.54±168.92	0.49
	Female	40	206.04±155.56	
CRP mg/l	Male	34	113.78±125.43	0.22
	Female	40	81.26±96.098	
Ferritin ng/ml	Male	34	1323.64±714.47	0.10
	Female	40	1034.48±776.91	
ESR mm/h.	Male	34	49.18±26.98	0.56
	Female	40	45.63±24.92	

All data represented by *t*-test: *P* value<0.05 (P*<0.05 significant), for comparing IL-6, Ilβ, TNF α, CRP, ferritin and ESR with the males and females.

CRP, C-reactive protein; ESR, erythrocyte sedimentation rate; IL-6, interleukin-6; IL-1β, interleukin-1-beta; TNF-α, tumour necrosis factor-alpha.

### IL-6, CRP, ferritin and ESR were significantly increased among severe cases of COVID-19

The comparison analysis by post hoc test among the groups in this study based on the biomarkers showed that CRP, ferritin, ESR, and IL-6 are significantly changed in COVID-19 patients and could be a promising biomarker among the patients [Table 3].

**Table 3:** Multiple comparisons and differences between healthy, mild, and severe COVID-19 infections about various biomarkers

Biomarker name	Control vs. mild (*P* value)	Control vs. severe (*P* value)	Mild vs. severe (*P* value)
CRP mg/l	0.543^ns^	0.0001****	0.0001****
Ferritin ng/ml	0.017*	0.0001****	0.0001****
ESR mm/h	0.002**	0.0001****	0.0001****
IL-6	0.015*	0.0001****	0.01**
IL1β	0.136^ns^	0.997^ns^	0.077^ns^
TNF-α	0.117^ns^	0.910^ns^	0.166^ns^

A Post Hoc test (Turkey HSD) was done to show the significance among the groups based on the biomarkers considered in this study.

CRP, C-reactive protein; ESR, erythrocyte sedimentation rate; IL-6, interleukin-6; IL-1β, interleukin-1-beta; ns, no significant; TNF-α, tumour necrosis factor-alpha.

*P* * < 0.05, *P* ** < 0.01, *P* ***< 0.001, **** *P* < 0.0001.

**Figure 1:**
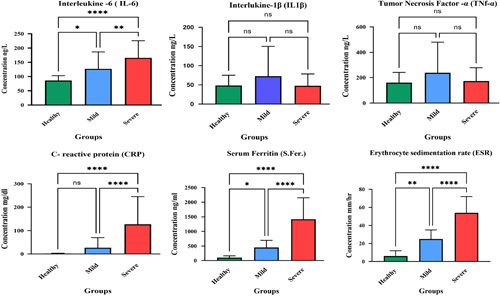
The levels of cytokines and biochemical tests in COVID-19 patients with different severity and controls group. Seventy-four COVID-19 patients were divided into two groups: mild and severe. The serum concentration of ferritin, CRP, TNF-α, IL-1β, and IL-6 were analyzed, and the ESR test was done, represented by *P* value: P***<0.001 (very highly significant), P**<0.01 (highly significant) and P* (Significant). CRP, C-reactive protein; ESR, erythrocyte sedimentation rate; IL-6, interleukin-6; IL-1β, interleukin-1-beta; TNF-α, tumour necrosis factor-alpha.

Serum IL-6 levels were increased in COVID-19 patients. The IL-6 concentrations in control, mild, and severe SARS-CoV-2 groups were (86.68±17.30), (127.12±59.56), and (165.85±60.13) ng/L, respectively. Compared with the control subjects, statistically significant differences were found for the mild and severe group patients, suggesting a positive relationship between the IL-6 concentration and the severity of COVID-19. The serum concentration of TNF-α and IL-1β levels did not change among COVID-19 patients. The TNF-α concentrations in control, mild, and severe SARS-CoV-2 groups were (160.18±81.20), (238.62±241.54), and (173.67±104.68) ng/L, respectively. There were no notable variations between the groups. The IL-1 concentrations were (48.4926.45), (72.3378.11), and (47.7830.42) ng/l in the control, mild, and severe SARS-CoV-2 groups, respectively. The SARS-CoV-2 groups’ levels did not significantly differ from those of the control group (Fig. 1).

In control, mild, and severe groups, the serum ferritin values were 123.80±93.39, 539.43±441.55, and 1450.51±699.64 ng/ml, respectively. Significant differences existed between the levels in the mild and severe groups and the control group. In the control, mild, and severe groups, the serum CRP concentrations were 2.34±1.45, 27.42±43.26, and 127.22±118.29 mg/l, respectively. The concentrations in the moderate group significantly elevated, while the levels in the severe groups were significantly different from those in the healthy control group. However, there is no significant difference from the levels in the healthy control group. The ESR levels were 7.73±4.05, 25.87±12.03, and 56.90 ±24.51 mm/h. In control, mild, and severe groups, respectively. The mild and severe groups’ levels differed significantly from those in the control groups, as shown in Figure 1.

### Relation between IL-6 with CRP, ferritin and ESR as a biomarker

**Figure 2:**
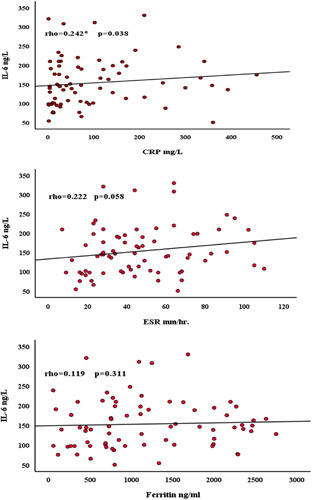
Scatter dot diagram represents the relationship between (IL-6 and CRP), (IL-6 and ferritin), (IL-6 and ESR). Spearman rank correlation analysis was performed to evaluate the correlation of serum IL-6 with three inflammatory markers in the patients with SARS-CoV-2 (mild and severe). CRP, C-reactive protein; ESR, erythrocyte sedimentation rate; IL-6, interleukin-6.

There was a significant difference in serum levels of CRP, ferritin, and IL-6 when the mild and severe cases were compared. Serum CRP was significantly higher in severe cases of COVID-19 compared to mild cases. The serum CRP levels showed an increase with the progression of the disease. Similar observations were seen about ESR, ferritin and IL-6. Because ferritin, ESR and CRP concentration was introduced as diagnostic biomarker for SARS-CoV-2 infections, so further analyzed the correlations between IL-6 concentration and CRP, ferritin and ESR amount. As shown in Figure 2, CRP concentration was positively correlated with IL-6 concentration (rho=0.242, *P*=0.038), also statistically significant within IL-6. Moreover, IL-6 concentration was also associated with ESR and ferritin levels, including (rho=0.222, *P*=0.058), (rho=0.119, *P*=0.311), respectively. However, the Analysis was not statistically significant (Fig. 2).

## Discussion

A rapid and well-coordinated innate immune response is the first line of protection against viral infection. Cytokines and chemokines have typically been suggested to play significant roles in immunopathology during viral infections like SARS-CoV-2^13^. Cytokines can be utilized to detect the cytokine storm while being expensive to test. Increases in proinflammatory cytokines, including IL-1β, IL-6, and TNF-α, are indicators of illness progression and an approaching cytokine storm^7^. Additionally, these cytokines can be utilized as prognostic markers to assess the efficacy of medications used to treat COVID-19 and evaluate the various stages of disease severity. This study aimed to look for the relationship between the severity of SARS-CoV-2 infection and using some proinflammatory cytokines with the biochemical test. As a result, it is stated by this study that IL-6 with CRP, ferritin and ESR could be a useful marker for detection the severe cases among SARS-CoV-2 infection because they altogether increased in comparison to non-infected individuals.

TNF-α and IL-6 rapidly rise in COVID-19 patients with severe symptoms and poor prognosis, but they quickly fall in COVID-19 individuals with milder symptoms^14^. Another important family of cytokines in cytokine storms is IL-1. Upregulation of IL-6 is one of the functions of IL-1β, an IL-1 family member. Consequently, the IL-1/IL-6/CRP axis is essential for developing inflammation^15^. Other investigations showed that the levels of several proinflammatory effector cytokines, including IL-1β, TNF-α, IL-6, IL-8, granulocyte-macrophage colony-stimulating factor, and granulocyte colony-stimulating factor, were elevated in SARS-CoV-2 infection as well as chemokines, such as IFN-induced protein 10 (IP10), Monocyte Chemoattractant Protein-1 (MCP-1), and Macrophage Inflammatory Proteins 1α (MIP1α, CCL3) are elevated in patients and associated with disease severity^16^. In this regard, our research has shown that the combination of serum ferritin, CRP, and ESR with a proinflammatory cytokine marker(IL-1β, IL-6, and TNFα) could be decisive in determining the severity of the SARS-CoV-2 infection. Patients in the severe group had significantly higher levels of IL-6, serum ferritin, CRP, and ESR than those in the mild and healthy groups. In contrast, patients in the different stages of disease severity and the healthy group did not differ significantly in their levels of the other two cytokines, IL-1β and TNF-α. In this research, specific inflammatory cytokines were examined as prospective biomarkers to detect virally caused inflammation and other biomarkers that indicated the severity of infections.

The retrospective study conducted by Pan and colleagues observed that patients mainly had higher serum concentrations of serum IL-6. Additionally, the plasma IL-6 concentration was noticeably greater in Intensive care unit (Severe case) patients than in non-ICU (Mild) patients. However, Interferon-gamma (IFN-γ), IL-17, IL-10, IL-4, and TNF-α were all almost within the normal range, consistent with our findings^17^. Our research closely supports the results of Chen *et al*.^18^, who found that in patients with severe disease, IL-6 levels increased but that IL-1, IL-8, and IL-10 concentrations did not change. Additionally, Gupta and colleagues showed that six cytokines (IL-10, TNF-α, IFN-γ, IL-4, IL-6, and IL-17A) expressions were assessed. Most of them were higher in COVID-19 individuals, although all of them except IL-6 levels were not statistically significant. Except for IL-6, which was statistically significant, increased severity did not translate into higher cytokine levels among the three COVID-19 severity groups^19^. Our research showed that IL-1β and TNF- α were not significantly different in different groups (mild and severe), which is totally in line with the Chinese study by Zhang and colleagues The levels between the Severe acute respiratory syndrome (SARS) groups and the healthy control group did not significantly differ. The IL-6 concentration increased in SARS patients and was significantly higher in those with the severe form of the disease, indicating a correlation between the serum IL-6 concentration and the severity of SARS^20^.

Chen and colleagues reported that in severe cases of COVID-19, there were higher levels of IL-6 and TNF-α than in mild cases. These authors also stated that levels of IL-1β were similar in severe and moderate cases^21^. This result diverges from our findings in TNF-α, which have not shown a different concentration level in various stages of the disease. A previous study by Bergantini reported no changes in TNF-α concentrations between COVID severity groups and controls. In contrast, the severe COVID-19 patient group, when compared with mild and healthy control groups, exhibited more significant amounts of IL-6 and IL-1β^22^. These results and relevant data from COVID-19 patients demonstrated that the immune system is compromised throughout the disease and that proinflammatory responses are crucial to the pathogenesis of SARS-CoV-2. According to a previous study, proinflammatory cytokines such (as IL-1, IL-6, IFN-γ, TNF-α, IL15, and IL-17) were present in higher concentrations in Middle East respiratory syndrome coronavirus patients^23^.

Meanwhile, a recent study indicated that patients with COVID-19 also showed elevated levels of cytokine profiles in their serum, including TNF-α, IL-1, IL-6, and IFN-γ^24^. Furthermore, in another study in the Iranian population, Taghiloo and his colleagues discovered that patients had higher levels of IFN-γ, IL-2, GM-CSF, IL-1β, IL-6, IL-8, IL-4, and TNF-α than the control group. Depending on clinical severity, all cytokine levels were higher in the severe cases than in the mild group; this finding is also clearly different from our findings^25^.

Ferritin and CRP are two more mediators of inflammation that are significantly elevated in severe conditions. Through Analysis of a large cohort of COVID-19 patients, Qin *et al*.^13^ demonstrated that the elevation of IL-6, IL-8, IL-10, CRP, and ferritin in patients with severe disease was statistically significant when compared with mild disease. Evidence suggests that ferritin can influence the immune response in chronic inflammation by upregulating anti-inflammatory cytokines and reducing free radical damage^26^. In addition, a recent study indicates that ferritin may play an essential role in the inflammatory pathophysiology of disease^26^. Acute-phase proteins that increase during infections, especially in COVID-19 patients, include the ferritin marker^27^. This coincides with our findings, which indicated that severe COVID-19 patients had greater ferritin levels than non-severe patients.

CRP is essential in several aspects of the inflammatory process. CRP participates in an innate immune response that causes complement activation and phagocytosis by attaching pathogens and damaged cellular components via phosphocholine. The liver cells release CRP in response to IL-6 stimulation. Because it responds so quickly to the inflammatory process and COVID-19 patients have dramatically higher levels, the acute-phase reactant CRP is crucial for these individuals^28,29^. The cytokine release syndrome can be accurately predicted by CRP, which can also be used to determine the severity of an illness. CRP levels that are abnormally high are directly associated with poor clinical prognosis^30^. It can be utilized as a surrogate marker because of the correlation between CRP levels and IL-6. It is crucial to observe patients daily to distinguish between patients whose fever would go away and those whose symptoms would turn into a cytokine storm. According to our findings, patients with severe and mild SARS-CoV-2 infections had significantly higher CRP levels than healthy individuals. This coincides with other results that indicated people with COVID-19 had more elevated CRP^28,31^.

The rate at which red blood cells settle in anticoagulated whole blood is measured by the inflammatory and immunological marker known as ESR^32^. Because the red blood cells stay together and become denser during inflammation due to a high level of fibrinogen in the blood, the ESR is faster than usual^33^. Variability in plasma viscosity may contribute to the aggregation of red blood cells^32^. ESR was typically higher in patients with coronaviruses than in healthy individuals. Our findings, which were in line with those of Lapić *et al*.^9^, demonstrated that ESR was more elevated in COVID-19 patients. Among COVID-19 patients, ESR, which is mainly tested along with CRP, was typically found to be high (15–85%)^34^ without any alteration indicated by the lung Computed tomographyscan, patients with severe and mild COVID-19 were found to have elevated CRP and ESR^35^. Predicting a poor prognosis for severe disease, ESR and CRP could be crucial and inexpensive monitoring parameters. Similar to a study by Han *et al*.^36^, our correlation analysis of IL-6 with CRP, ESR, and ferritin among COVID-19 patients revealed that only IL-6 was significantly positively correlated with CRP, as shown in Figure 2. In addition, our result concurs with Taghiloo *et al*.^25^, who demonstrated that IL-6 was significantly positively correlated with CRP concentration.

Genetic, social, cultural, and behavioural tendencies form the complicated epidemiological concept of ethnicity. Despite the lack of ethnicity factors in COVID-19, there is persistent evidence that ethnic minorities have higher infection rates^37^. Nevertheless, there is still some uncertainty regarding the connection between racial background and the SARS-CoV-2 infection^38^. Our study of the Kurdish ethnic group in the Sulaimani city of Iraq is closely related to studies by Merza and colleagues of the Kurdish ethnic group in the Erbil city of Iraq, who showed an increase in IL-6 levels but no change in TNF-α or IL-1 concentration in patients with severe disease. Both studies were conducted among members of the Kurdish ethnic group in different geographic locations, making them closely comparable^39^. As all of the participants in our study were Kurdish citizens of Iraq, it was difficult to generalize our findings to other ethnic groups. According to a prospective study by Can *et al*.^40^, among Turks, these markers can be used to indicate the severity of COVID-19 since they show an increase in CRP, ESR, and serum ferritin levels in the severe patient group.

Furthermore, in another study in the Iranian population, Taghiloo and his colleagues discovered that patients had higher levels of IL-1β, IL-6, and TNF-α than the control group. Depending on clinical severity, all cytokine levels were higher in the severe cases than in the mild group^25^. The study by Chi *et al*.^41^, conducted among china ethnic group, indicated that the serum IL-1β, IL-6, and TNF-α levels were higher in symptomatic patients (mild, moderate, Severe) when compared with healthy individuals. The prevalence of comorbid conditions like diabetes, hypertension, and chronic kidney disease, as well as the accompanying symptoms like fever, coughing, or shortness of breath at the time of admission for COVID-19 patients, were all found to vary in different ethnic populations by Price-Haywood and colleagues, They also noticed that there were considerably higher levels of biomarkers for cardiac damage, liver, kidney, and inflammation as well as coagulation. These factors impacted how different locations had differing rates of COVID-19 positive, hospitalization, intensive care, and mortality^42^. Various studies on proinflammatory cytokines and other inflammatory markers that are important for determining the severity of disease have found that different ethnic groups have different immune responses. As a result, our findings might not apply to all ethnic groups, cities, or countries in the world. To better understand how ethnicity affects COVID-19 illness features, more study is needed

Some limitations are considered in this study. First, it was a case-control study with a small sample size. Second, due to the expense and lack of funding, we could not examine more significant recently identified cytokines helpful for illness severity and diagnosis. Third, we could not include an equal number of men and women in our study. Fourth, we could not control drug users before they entered the hospital since all patients used classes of medications such (as azithromycin, vitamin D, hydrchloroquin, betamethasone, and paracetamol), which may impact the range of proinflammatory cytokines.

## Conclusion

Comparing different groups of COVID-19 patients with a healthy control group, the levels of IL-6, serum ferritin, CRP, and ESR demonstrated significant differences. As a result, for COVID-19 patients, all four levels have a high potential for outcome prediction. The findings of this study, as the first study in the cities of Sulaymaniyah in Iraq, suggest that the levels of IL-6, ferritin, ESR, and CRP could be used to predict the severity of COVID-19 disease. Finally, it may be helpful for management and treatment during the time course of the disease. We proposed that additional studies were required to identify the variables that affected and contributed to the overproduction of proinflammatory cytokines, such as single nucleotide polymorphisms in the promoter region of proinflammatory genes among Kurdish people in the Sulaymaniyah region of Iraq, the epigenetic factor that contributes to the issue at various stages of covid-19 diseases, and varied host immune response in distinct populations.

## Ethics approval

Ethical approval for the present study was received from the College of Medicine/Sulaimani University-Sulaymaniyah ethical committee (75 on 19 June 2022).

## Consent to participate

Not applicable.

## Consent for publication

Not applicable.

## Source of funding

S.S.F. and P.J.J. supported this study.

## Authors contribution

S.S.F.: data acquisition, doing the experiments and analysis. P.J.J. wrote the main manuscript and revised the study. All authors read and approved the final manuscript.

## Conflicts of interest disclosure

The authors declare no competing interests

## Provenance and peer review

Not commissioned, externally peer-reviewed.
